# Investigating Non-sterilizing Cure in TB Patients at the End of Successful Anti-TB Therapy

**DOI:** 10.3389/fcimb.2020.00443

**Published:** 2020-08-25

**Authors:** Caroline G. G. Beltran, Tiaan Heunis, James Gallant, Rouxjeane Venter, Nelita du Plessis, Andre G. Loxton, Matthias Trost, Jill Winter, Stephanus T. Malherbe, Bavesh D. Kana, Gerhard Walzl

**Affiliations:** ^1^Department of Science and Technology/National Research Foundation, Centre of Excellence for Biomedical Tuberculosis Research and South African Medical Research Council Centre for Tuberculosis Research, Cape Town, South Africa; ^2^Division of Molecular Biology and Human Genetics, Department of Biomedical Sciences, Faculty of Medicine and Health Sciences, Stellenbosch University, Cape Town, South Africa; ^3^Faculty of Medical Sciences, Biosciences Institute, Newcastle University, Newcastle upon Tyne, United Kingdom; ^4^Section Molecular Microbiology, Amsterdam Institute for Molecules, Medicines and Systems, Vrije Universiteit Amsterdam, Amsterdam, Netherlands; ^5^Catalysis Foundation for Health, San Ramon, CA, United States; ^6^DST/NRF Centre of Excellence for Biomedical TB Research, Faculty of Health Sciences, School of Pathology, University of the Witwatersrand and the National Health Laboratory Service, Johannesburg, South Africa; ^7^MRC-CAPRISA HIV-TB Pathogenesis and Treatment Research Unit, Centre for the AIDS Programme of Research in South Africa, CAPRISA, Durban, South Africa

**Keywords:** tuberculosis, sterilizing cure, *Mycobacterium tuberculosis*, differentially culturable tubercle bacteria, dormant, 18F-FDG PET-CT, BALF proteome analysis, treatment response

## Abstract

*Mycobacterium tuberculosis* (Mtb) is extremely recalcitrant to antimicrobial chemotherapy requiring 6 months to treat drug-sensitive tuberculosis (TB). Despite this, 4–10% of cured patients will develop recurrent disease within 12 months after completing therapy. Reasons for relapse in cured TB patients remains speculative, attributed to both pathogen and host factors. Populations of dormant bacilli are hypothesized to cause relapse in initially cured TB patients however, development of tests to convincingly demonstrate their presence at the end of anti-TB treatment has been challenging. Previous studies have indicated the utility of culture filtrate supplemented media (CFSM) to detect differentially culturable tubercle bacilli (DCTB). Here, we show that 3/22 of clinically cured patients retained DCTB in induced sputum and bronchoalveolar lavage fluid (BALF), with one DCTB positive patient relapsing within the first year of completing therapy. We also show a correlation of DCTB status with “unresolved” end of treatment FDG PET-CT imaging. Additionally, 19 end of treatment induced sputum samples from patients not undergoing bronchoscopy were assessed for DCTB, identifying a further relapse case with DCTB. We further show that induced sputum is a less reliable source for the DCTB assay at the end of treatment, limiting the utility of this assay in a clinical setting. We next investigated the host proteome at the site of disease (BALF) using multiplexed proteomic analysis and compared these to active TB cases to identify host-specific factors indicative of cure. Distinct signatures stratified active from cured TB patients into distinct groups, with a DCTB positive, subsequently relapsing, end of treatment patient showing a proteomic signature closer to active TB disease than cure. This exploratory study offers evidence of live Mtb, undetectable with conventional culture methods, at the end of clinically successful treatment and putative host protein biomarkers of active disease and cure. These findings have implications for the assessment of true sterilizing cure in TB patients and opens new avenues for targeted approaches to monitor treatment response.

## Introduction

*Mycobacterium tuberculosis* (Mtb) is a highly complex and well-adapted pathogen that causes tuberculosis (TB). The success of this pathogen can be attributed to its ability to evade the protective host immune response and its recalcitrance to antimicrobial chemotherapy. Although cure of the majority of patients treated with the standard 6 month multidrug regimen indicates that treatment is highly effective, ~4–10% of clinically cured patients will develop recurrent disease within the first 12 months after completing therapy (Naidoo and Dookie, 2018; World Health Organization, 2019). Recurrence can fall into two categories; (1) relapse due to the reactivation of the original infecting strain or (2) reinfection due to exogenous infection with a strain of Mtb different from the original infecting strain (Naidoo and Dookie, 2018). In high endemic countries, the line between reinfection and relapse is often blurred since patients are likely to be continually exposed to similar strains (Warren et al., 2004).

Current observed relapse rates indicate that a fraction of patients, whilst appearing clinically cured, still harbor a sub-clinical Mtb infection that failed to be sterilized by current treatment regimens. Indeed, there is growing evidence pointing to residual live Mtb in patients at the end of treatment (Malherbe et al., 2016; Ambreen et al., 2019). Previous work from our group has shown that most patients at the end of treatment retain 18F-fluorodyoxyglucose positron emission tomography (FDG-PET) avid lung lesions indicative of persisting inflammation, even after sputum cultures are consistently negative. Furthermore, Mtb mRNA can be detected in sputum and bronchoalveolar lavage fluid (BALF) in a significant fraction of cured patients at the end of curative treatment, possibly indicating residual live Mtb (Malherbe et al., 2016). It is well-established that Mtb, amongst other bacteria, can form physiologically heterogeneous populations both *in-vitro* and *in-vivo* (Balaban et al., 2004; Gefen and Balaban, 2009; Lewis, 2010; Manina et al., 2015; Walter et al., 2015; Fisher et al., 2017). In patients, a sub-population of metabolically distinct bacilli, defined as differentially culturable tubercle bacilli (DCTB) can be detected in sputa when grown in the presence of sterilized culture filtrate supplemented media (CFSM). This sub-population appears to become more prominent during early chemotherapy (Mukamolova et al., 2010; Chengalroyen et al., 2016), yet is not directly detectable by conventional microbiological diagnostic methods. It remains to be conclusively determined whether the presence of DCTB cells correlates with clinical treatment response and relapse rates. Host-specific factors are likely to also be critical in determining favorable or unfavorable outcomes even in the presence of persistent bacteria.

Part of the complexity of the problem lies with the host itself, where, paradoxically, anatomic niches in the form of granulomas sequester infectious bacteria from the lung and favor the development and long-term survival of dormant Mtb, subsequently giving rise to actively replicating bacterial populations and dissemination (Russell et al., 2009; Gengenbacher and Kaufmann, 2012; Ehlers and Schaible, 2013; Gollan et al., 2019). The link between dormancy and reactivation of disease has been demonstrated in animal models for several bacterial species, including Mtb (McCune et al., 1956, 1966a,b; Griffin et al., 2011; Kaiser et al., 2014).

The aim of this study was to investigate whether patients at the end of successful anti-TB treatment retain live, DCTB and to correlate this with FDG PET-CT activity. We used CFSM to culture mycobacteria from both BALF and induced sputum. We also analyzed BALF supernatant from these same clinically cured patients and an equal number of active TB samples, using shotgun proteomics. The results provide a global picture of the host proteome at the site of disease and gives insight into host responses to active TB infection vs. clinical cure. The finding that DCTB and host biomarkers for active TB may be present in cured TB patients, albeit in a small proportion of patients, opens new avenues for targeted approaches to monitor treatment response.

## Methods

**Figure 1:**
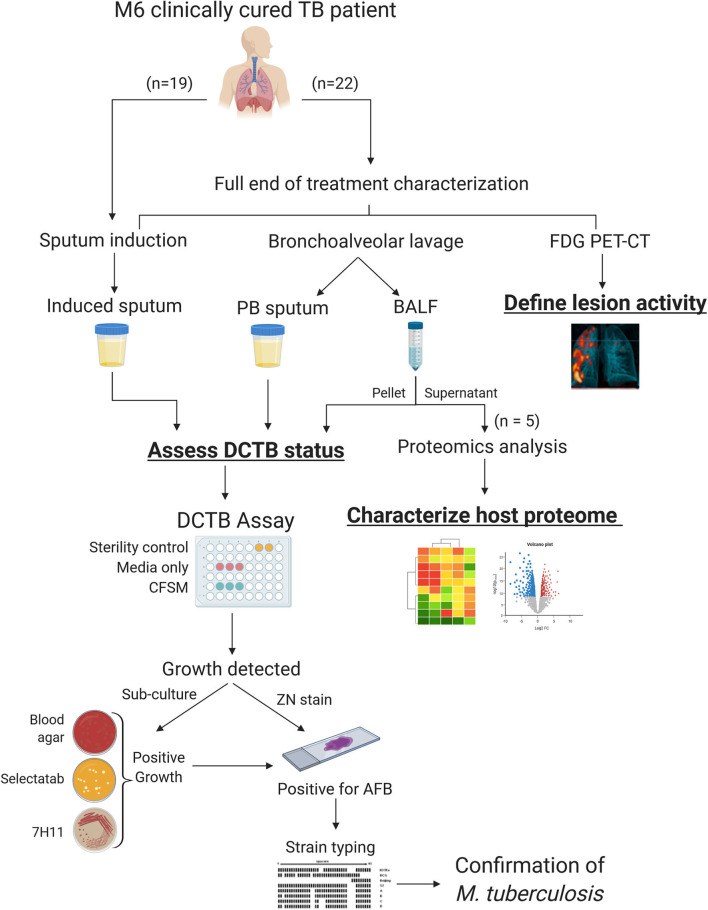
Sample processing and workflow used to investigate sterilizing cure in tuberculosis (TB) patients at the end of successful anti-TB therapy. Three sample types were analyzed for DCTB *M. tuberculosis* in clinically cured end of treatment patients (*n* = 22); an induced sputum, bronchoalveolar lavage fluid (BALF) and post-bronchoscopy (PB) sputum (*n* = 22). These patients were also scanned using FDG PET-CT to classify lesion activity at the end of treatment. The BALF supernatant from five patients was used for multiplexed proteomics analysis using tandem mass tag (TMT 10 plex) and compared to the proteome of active TB cases. A further 19 EOT patients were assessed for DCTB in their induced sputum, only. The DCTB assay consisted of a series of confirmatory tests once growth was detected including sub-culturing on blood agar, *Mycobacterium* Selectatab and Middlebrook 7H11 Agar. Colonies corresponding to typical growth of *M. tuberculosis* were subsequently picked and confirmed for acid fast bacilli (AFB) using ZN staining before being strain typed using spoligotyping.

A summary workflow diagram of the sample recruitment and methods is presented in Figure 1. Twenty-two patients were included for a full characterization at the end of treatment (EOT). Patients were scanned by FDG PET-CT imaging and had a sputum induction by inhalation of hypertonic saline. These patients also underwent a bronchoscopy where a bronchoalveolar lavage fluid (BALF) and post-bronchoscopy sputum (PB sputum) was collected. The induced sputum, PB sputum and BALF pellet were assessed for DCTB at EOT and to correlate this with FDG PET-CT lesion activity. A subset of the BALF supernatant samples (*n* = 5) were used to develop a protocol that utilized proteomics analysis to provide insight into the global host proteomics changes at the site of disease. A further nineteen EOT patients were assessed for DCTB in their induced sputum, only.

### Patient Recruitment

Ethical consent was obtained from the Medical Human Research Ethics committee (HREC) of Stellenbosch University (HREC references: N10/01/013 and N16/05/070). All methods were performed in accordance with the relevant guidelines and regulations. EOT patients were included in the study if they met the following criteria: willing to give consent, willing to have their HIV status tested, aged between 18 and 70 years, not taking any corticosteroid medication, having undergone successful anti-TB treatment (confirmed by two consecutive, negative sputum cultures). Detailed clinical medical history was taken at baseline (history of smoking, previous TB, other medication) and physical tests included chest X-rays and blood test for HbA1c level. All patients were given standard anti-TB treatment consisting of 2 months intensive phase [rifampicin (RIF), isoniazid (INH), ethambutol and pyrazinamide] and 4 months of RIF and INH as continuation phase. After informed consent, 41 EOT patients were recruited from studies that took place in primary health care clinics in Northern Cape Town. Patients were followed up to 1 year after the end of treatment (M18) to assess recurrence. Ten healthy house-hold contacts (negative controls) from the same community and two pretreatment active TB cases (positive controls) were included as control subjects for the DCTB assay. A further five pretreatment active TB cases were recruited for proteomics analysis.

### End of Treatment Induced Sputum

Sputum was induced with 20 mL of 3% hypertonic saline solution delivered through ultrasonic nebulizer and collected in a sterile sputum container. Sputum samples were stored at 4°C prior to processing (sputa were processed within 24 h of collection to minimize growth of commensal bacteria).

### FDG PET-CT

Patients were scanned at the Western Cape Academic Positron Emission Tomography (PET) Center, at Tygerberg Academic Hospital. All patients received between 5 and 7 mCi of intravenous 18F FDG (18-F Fluorodeoxyglucose), which is taken up by metabolically active cells and indicates inflammation. Patients were scanned 1 h after the injection using a Philips Gemini scanner. Whole lung analysis was performed on the scans to quantify (1) the total glycolytic activity index (TGAI) on PET (indicating total inflammatory burden) using lesion-free lung as a background (Malherbe et al., 2018, 2020), (2) the most intense residual lesion uptake on PET, (3) the total residual cavity volume on CT.

### EOT Bronchoscopy and BALF Collection

One week following the FDG PET-CT scan and induced sputum visit, an experienced bronchoscopist conducted a bronchoscopy and bronchoalveolar lavage on patients at the Pulmonology Unit, Tygerberg Academic Hospital. Up to 200 mL of sterile physiologic saline solution were instilled (in divided aliquots of 20–60 mL each) and subsequently aspirated to obtain BALF from the lobe that showed the most residual infiltrates on an accompanying chest X-ray. BALF and the induced sputum samples were split into two volumes following collection and immediately processed. A post-bronchoscopy (PB) sputum was collected immediately after the bronchoscopy for the DCTB assay.

### Modified DCTB Assay

#### Sputum and BALF Decontamination

BALF samples (50 mL) were concentrated by centrifugation at 4,000 × g for 20 min at 10°C, the supernatant transferred to a fresh tube and stored at −80°C for proteomics analysis, leaving ~5 mL pellet. The BALF pellets and sputa were decontaminated by adding equal volumes of N-acetyl-L-cysteine- 2% sodium hydroxide (NALC-NaOH) using the commercial MycoPrep™ kit (BD Biosciences, USA) and incubating for 15 min at room temperature (RT) with brief vortexing every 5 min. Specimens were neutralized with phosphate buffer saline (PBS) (33 mM Na_2_HPO_4_, 33 mM KH_2_PO_4_; pH 6.8) and concentrated by centrifugation at 4,000 × g for 20 min at 10°C. The pellets were resuspended in 300 μL PBS and used immediately for the DCTB assay.

#### Preparation of Culture Filtrate Supplemented Media

The laboratory strain of Mtb, H37Rv, was used to produce culture filtrate (CF), as described previously (Loraine et al., 2016). Briefly, Mtb cultures were grown in Middlebrook 7H9 medium containing 0.05% tween and supplemented with 10% OADC (oleic acid, albumin, dextrose, and catalase; BD Biosciences) in vented flasks and kept stationary at 37°C until mid-exponential phase was reached (OD600 nm 0.6–1.0). Cultures were centrifuged at 4,000 × g for 15 min and supernatants were sterilized by double filtration using sterile, disposable Durapore Membrane Filters PVDF (0.22 μm pore size). CF was supplemented with fresh 7H9 media (at a 1:1 ratio) and polymyxin B, amphotericin B, nalidixic acid, trimethoprim, and azlocillin (PANTA™ antibiotic mixture, BD Biosciences) were added to increase subsequent selectivity for Mtb growth. A 450 μL aliquot of the CFSM was added to a 48-well cell culture plate (Nunc, Thermo Scientific). Sterility of the CF was verified by including neat aliquots in control wells of the cell culture plate.

#### Addition of Sample to 48-Well Culture Plate

Decontaminated induced sputum and BALF samples (50 μL) were added in triplicate to each well containing CFSM. An additional three wells containing 7H9 media (without CFSM) were also included as a media control comparison and the same volume of samples was added to each of these wells (see DCTB assay for plate layout in Figure 1). For the blank controls, 50 μL of PBS was added to the CFSM. Culture plates were sealed with micropore tape and incubated without shaking at 37°C for a period of 8 weeks. Plates were checked for growth weekly.

#### Confirmation of *M. tuberculosis* Growth

Confirmatory assays were conducted once growth was identified visually (through observation of turbidity), to confirm the presence of Mtb, as opposed to contamination by other microorganisms. Confirmatory testing included a combination of Ziehl-Neelsen (ZN) staining and sub-culturing onto; (1) blood agar plates to check for contamination; (2) *Mycobacterium* Selectatab media (Mast Group) to isolate single colonies and (3) Middlebrook 7H11 Agar (BD Life Sciences). Once colonies corresponding to typical growth of Mtb (non-pigmented, rough, dry colonies) were detected on Selectatab media and/or 7H11 media, a colony was picked for ZN staining as well as resuspended in 500 μL ultrapure water (MilliQ, Merck) and heat inactivated at 100°C for 30 min to extract DNA for strain typing using spoligotyping. Replicate wells for both CFSM and media control were followed up independently. A negative culture well was defined as one in which neither Mtb grew, nor contamination was observed. Spoligotyping was conducted as previously described (Streicher et al., 2007), and the recovered strain was matched to the original infecting strain isolated from baseline sputum cultures.

### Proteomics Analysis

#### Samples

For the proteomics analysis of BALF supernatant, five EOT patients also analyzed for DCTB (Figure 1), were chosen (including PID338 who subsequently relapsed) and BALF from five active TB (before treatment initiation) patients were included as a comparison. The active TB patients were recruited at their baseline visit and underwent a bronchoscopy, as described above. After cell collection, the BALF supernatants (15 mL) were filter sterilized using 0.22 μm PVDF filters prior to processing. The filtered BALF samples were concentrated using Amicon ultra-15 kDa centrifugal filters (Merck) to ~2.5 mL and depleted using the ProteoPrep Blue Albumin and IgG Depletion kit (Sigma-Aldrich), as recommended by the supplier. Ice-cold acetone was added to the depleted samples and proteins were precipitated overnight at −20°C. Samples were centrifuged at 14,000 × g for 10 min and the supernatant removed. The protein pellets were resuspended in 50 μL 8 M urea in 100 mM triethylammonium bicarbonate (TEAB) and sonicated on ice for 10 min to completely reconstitute the protein. Protein concentration was determined using a Bradford assay.

#### Proteolytic Digestion and TMT Labeling of Peptides

Aliquots corresponding to 50 μg protein from each sample were used for filter-aided sample preparation (FASP) (Wiśniewski et al., 2009). Samples were reduced with 5 mM tris(2-carboxyethyl) phosphine (TCEP) for 1 h at RT and alkylated with 5.5 mM iodoacetamide (IAA) for 1 h in the dark at RT. Samples were concentrated using Amicon 30 kDa Ultra 0.5 mL spin filters (Merck) at 14,000 × *g* for 15 min before adding 400 μL 50 mM TEAB to each spin filter, and further centrifugation at 14,000 × *g* for 15 min. This process was repeated a further three times, discarding the flow through between washes. Following the wash steps, 400 μL of 50 mM TEAB was added directly onto each filter and the samples centrifuged at 14,000 × *g* for 15 min. This process was repeated a further two times. Modified sequencing grade trypsin (Promega) was added at a 1:50 ratio of trypsin to protein to each filter and samples were incubated at 37°C for 18 h. Following trypsin digestion, filters were transferred to a new collection tube and centrifuged at 14,000 × *g* for 15 min, collecting peptides in the flow through. Peptides were further eluted using 400 μL of 100 mM sodium chloride (NaCl) and centrifuged again. Peptide eluates were pooled and concentrated by vacuum drying. Peptides were subsequently desalted using in-house packed C18 STAGE tips, vacuum dried, and stored until labeling with tandem mass tags. TMT 10 plex (Thermo Fisher Scientific) labeling was done according to manufacturer's instructions. Briefly, peptide samples were reconstituted in 100 μL 100 mM TEAB and the TMT labels were reconstituted in 41 μL anhydrous acetonitrile. The labeling reaction was quenched using 8 μL of 5% hydroxylamine and incubating for 15 min at RT. Approximately 15 μg from each sample was combined into a new tube and desalted and fractionated using a C18 STAGE tip. STAGE tip-based fractionation was done using increasing gradients of acetonitrile in 10 mM TEAB as follows; 7.5, 10, 12.5, 15.5, 17.5, 20, 22.5, 25, 30, 50% and collecting the eluates between each gradient into individual tubes before vacuum drying.

#### Mass Spectrometry

Peptides were dissolved in 2% acetonitrile containing 0.1% trifluoroacetic acid, and each sample was independently analyzed on an Orbitrap Fusion Lumos Tribrid mass spectrometer (Thermo Fisher Scientific), connected to an UltiMate 3000 RSLCnano System (Thermo Fisher Scientific). Peptides were injected on an Acclaim PepMap 100 C18 LC trap column (100 μm ID ×20 mm, 3 μm, 100 Å) followed by separation on an EASY-Spray nanoLC C18 column (75 μm ID ×500 mm, 2 μm, 100 Å) at a flow rate of 300 nl.min^−1^. Solvent A was water containing 0.1% formic acid, and solvent B was 80% acetonitrile containing 0.1% formic acid. The gradient used for analysis was as follows: solvent B was maintained at 3% for 5 min, followed by an increase from 3 to 35% B in 90 min, 35–90% B in 0.5 min, maintained at 90% B for 4 min, followed by a decrease to 3% in 0.5 min and equilibration at 3% for 10 min. The Orbitrap Fusion Lumos was operated in positive-ion data-dependent mode. Precursor ion (MS1) scans were performed in the Orbitrap mass analyzer in the range of 375–1,500 m/z, with a resolution of 120,000 at 200 m/z. An automatic gain control (AGC) target of 4 × 10^5^ and an ion injection time of 50 ms was allowed. Precursor ions were isolated using a quadrupole mass filter with an isolation width of 0.7 m/z, and fragmented using collision-induced dissociation (CID) with a collision energy of 35%. MS2 spectra were acquired in the linear ion (IT) trap using turbo mode. An AGC target of 1 × 10^4^ and a maximum injection time of 50 ms was allowed. Ten MS2 fragment ions were co-selected for MS3 analysis using synchronous precursor selection (SPS), and underwent high-energy collisional dissociation (HCD) with collision energy set to 65% to ensure maximal TMT reporter ion yield. SPS-MS3 fragment ions were analyzed in the Orbitrap mass analyzer at a resolution of 60,000 at 200 m/z. An AGC target of 5 × 10^4^ and maximum injection time of 120 ms was allowed. The number of MS2 and MS3 events between full scans was determined on-the-fly to maintain a 3 s fixed duty cycle. Dynamic exclusion of ions within a ±10 ppm m/z window was implemented using a 35 s exclusion duration. An electrospray voltage of 2.0 kV and capillary temperature of 275°C, with no sheath and auxiliary gas flow, was used.

#### Mass Spectrometry Data Analysis

All spectra were analyzed using MaxQuant 1.6.2.6 (Cox and Mann, 2008), and searched against a combined *Homo sapiens* and Mtb database. Proteome databases were downloaded from Uniprot on 09 January 2019. The *Homo sapiens* database contained 42,410 entries while the Mtb database contained 3,993 entries. Peak list generation was performed within MaxQuant and searches were performed using default parameters and the built-in Andromeda search engine (Cox et al., 2011). The enzyme specificity was set to consider fully tryptic peptides, and two missed cleavages were allowed. Oxidation of methionine and deamidation of asparagine and glutamine was allowed as variable modification, while carbamidomethylation of cysteine was allowed as a fixed modification. TMT labeling (TMT 10 plex) of peptide N-termini and lysine residues was enabled during the database search. A protein and peptide false discovery rate (FDR) of <1% was employed in MaxQuant. Proteins that contained similar peptides and that could not be differentiated based on MS/MS analysis alone were grouped to satisfy the principles of parsimony. Reporter ion quantification (TMT 10 plex) at MS3-level was enabled in MaxQuant and reporter ion intensities were used for relative quantification. Reverse hits, contaminants, and proteins only identified by site were removed before further analysis.

Samples were grouped in either end of treatment (EOT) or active tuberculosis (TB) groups in accordance with sampling for this study. The corrected reporter ion intensities were log2 transformed and centered around zero by median normalization. Protein groups were further filtered to contain at least two unique peptides and to contain intensities for all the TMT-10 plex reporter ions before downstream exploratory, statistical, and bioinformatics analysis. Next, principal component analysis was performed to identify possible clusters in the data not based on any a priori knowledge. One of the EOT cases clustered with the active TB cases, indicating a protein signature similar to active TB. Follow up analysis revealed that this participant showed signs of active TB (modified culture confirmed) following successful anti-TB treatment and subsequently relapsed. Therefore, this EOT case was assigned as a TB case during statistical analysis. Hypothesis testing was performed using the Limma package (Ritchie et al., 2015), available in the R/Bioconductor repository (Gentleman et al., 2004), and *p*-values were corrected for multiple hypothesis testing using the Benjamini-Hochberg method (Gentleman et al., 2004; Ritchie et al., 2015). Proteins were considered significantly regulated when they had at least a two-fold change difference between groups and a corrected *p*-value <0.05. Enrichment analysis was performed using ClusterProfiler (Yu et al., 2012) and ReactomePA (Yu and He, 2015), and Gene Set Enrichment analysis (GSEA) was performed using WebGestalt (Liao et al., 2019).

## Results

### Differentially Culturable Mtb Are Present at the End of Treatment

**Figure 2:**
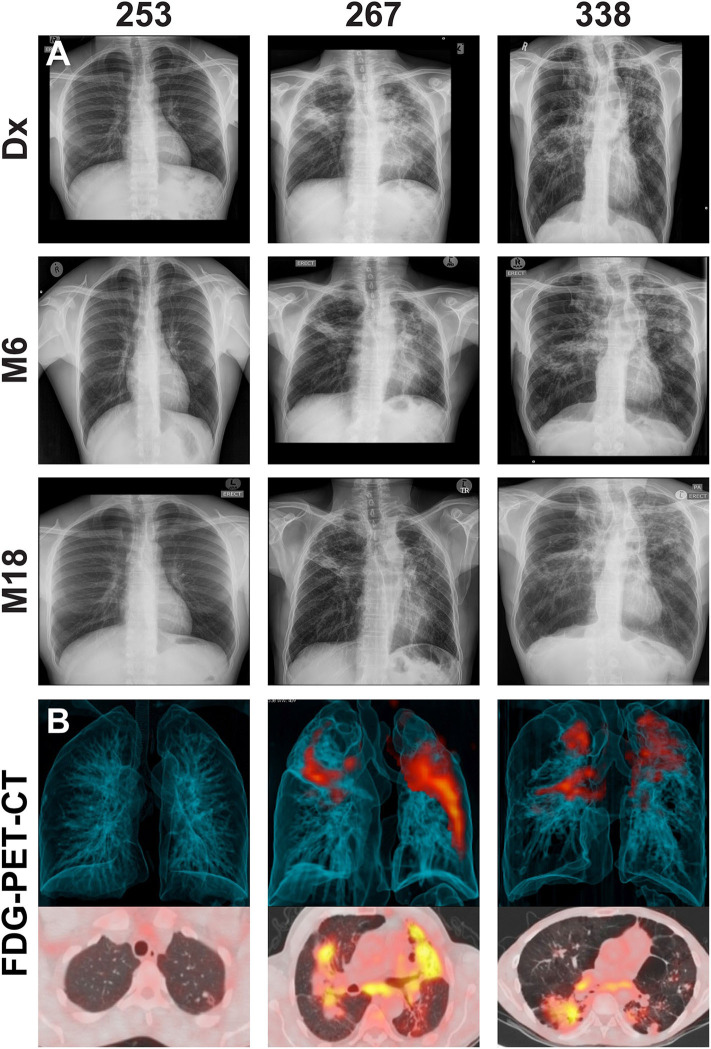
**(A)** Representative chest X-rays at diagnosis (Dx), month 6 (M6) and month 18 (M18) and **(B)** corresponding FDG PET-CT scan at M6 for cured patients with differentially culturable Mtb. The FDG PET-CT scans show 3-dimensional anterior (top panel) and transverse (lower panels) views.

There were 22 clinically cured patients (negative sputum MGIT cultures after 20 and 24 weeks of anti-tuberculous treatment) analyzed for FDG PET-CT activity recruited for assessment of DCTB in sputum and BALF (Table 1). Three patients (13.6%) had a positive DCTB status with one of the DCTB positive patients relapsing within the first year following treatment completion. FDG PET-CT scans indicated three main response patterns at the end of treatment: (1) A “resolved” PET response, seen in six (27.3%) patient scans, showing no residual increase in FDG uptake at the end of treatment. (2) An “improved response” type shows residual uptake (but decreased in comparison to diagnosis) in 11 patients (50%). (3) A “mixed response” (characterized by either new lesions or increased uptake in some lung lesions) in five patients (22.7%), including two of the three patients with DCTB (Table 1). Images of the M6 FDG PET-CT scan and chest X-rays at diagnosis (Dx), M6 and M18 follow up of the patients with DCTB are shown in Figure 2. In addition to the set of 22 patients with sputum and BALF samples, a further 19 clinically cured patients were recruited for assessment of DCTB in their induced sputum only, identifying a further two DCTB positive patients with one of these subsequently relapsing within a year of completing therapy (Table 2). The clinical and microbiological data for all 41 EOT patients is presented in Table S1. Treatment adherence was >96% for all patients and 75% of patients reached their first consecutive negative MGIT 8 weeks into treatment.

**Table 1:** Outcomes of patients assessed by FDG PET CT and the differentially culturable tubercle bacilli (DCTB) assay at month 6 after successful anti-TB therapy.

**PID**	**DCTB**	**Outcome**	**Response pattern**	**Intensity rank**	**TGAI**	**Total cavity volume (mL)**
338	POS	Relapse	Mixed	Very high	43007.0	39.9
267	POS	Cured	Mixed	Very high	38304.0	30.9
253	POS	Cured	Resolved	Minimal	70.36	0.0
Median (range)					38304.00 (70.4–43007)	30.9 (0–39.9)
332		Cured	Mixed	High	4598.2	0.0
351		Cured	Mixed	Moderate	2853.4	0.0
250		Cured	Mixed	Very high	620.0	62.9
334		Cured	Improved	Very high	9757.8	19.4
375		Cured	Improved	Very high	9562.6	1.8
291		Cured	Improved	Moderate	6349.8	5.9
392		Cured	Improved	Very high	6109.6	0.0
382		Cured	Improved	Very high	2795.4	2.8
335		Cured	Improved	High	2265.7	15.7
357		Cured	Improved	Moderate	634.9	0.0
278		Cured	Improved	Mild	585.6	0.0
315		Cured	Improved	Moderate	235.5	0.0
300		Cured	Improved	High	173.2	0.0
314		Cured	Improved	Mild	151.7	0.0
273		Cured	Resolved	None	1681.9	0.0
365		Cured	Resolved	Minimal	951.7	0.0
318		Cured	Resolved	Minimal	306.2	0.5
306		Cured	Resolved	Minimal	213.8	0.0
359		Cured	Resolved	Minimal	168.2	2.8
Median (range)					793.27 (151.7–9757.8)	0.0 (0.0–62.9)
			22.7% mixed			
			50% improved			
			27.3% resolved			

*PET CT responses were classified according to total glycolytic activity (TGAI), cavity, response pattern, and intensity rank. Highlights in red show patients classified as higher risk according to PET CT cut offs (TGAI ≥ 600, cavity volume ≥ 7 mL, mixed response, and very high intensity rank)*.

**Table 2:** Clinical and microbiological data for the five clinically cured patients positive for DCTB and outcome following 12 months follow up after cure.

**PID**	**Age**	**Sex**	**HIV**	**Hba1c**	**Smoker**	**Previous TB**	**Treatment adherence (%)**	**BMI Dx**	**BMI M6**	**CXR M6**	**CXR M18**	**DCTB**	**Source**	**Outcome**
253	29	M	NEG	5.9	Daily	NO	100	19.2	21.3	Worse	Improved	POS	BLF	Cured
267	57	M	NEG	6	EX	YES	100	18.2	21	No change	No change	POS	BLF/iS	Cured
338	48	M	NEG	5.3	Daily	YES	99	16	16.5	No change	No change	POS	BLF/iS/pbS	Relapse
370	26	F	NEG	5.2	< daily	NO	100	16.5	18.6	Improved	No change	POS	iS	Cured
404	49	F	NEG	NA	NO	YES	99	16.6	15.6	No change	No change	POS	iS	Relapse

*Source refers to DCTB found in either BALF (BLF), induced sputum (iS) or post-bronch sputum (pbS)*.

### BALF Is the Optimal Sample Type to Identify Differentially Culturable Mtb at the End of Treatment

The DCTB assay indicated that a significant portion of culture wells were positive for growth in all samples analyzed by visual inspection [induced sputum (81.8%), post-bronchoscopy sputum (54.5%) and BALF (41%)] (Table 3). However, the majority of these did not contain Mtb, as determined by confirmatory assays. Only samples that could be sub-cultured onto selective media and subsequently strain typed were declared positive for DCTB. As shown in Table 3, the number of false positives was highest in the induced sputum and lowest in BALF. The relapse patients (in samples taken before relapse) showed pure Mtb growth, without obvious contamination by commensal bacteria, in all sample types tested (BALF, induced and post-bronchoscopy sputum for PID338 and induced sputum for PID404) (Figure S1) and these grew rapidly in the micro-well format (<5 days). BALF was also the sample in which we could consistently detect all DCTB positive patients when comparing to induced sputum and post-bronchoscopy sputum (Table S1). Differential cell staining showed that sputa is comprised primarily of epithelial cells, whereas the cell profile of BALF samples is comprised of macrophages, lymphocytes, and neutrophils (Figure S2).

**Table 3:** Differentially culturable tubercle bacilli (DCTB) assay results comparing growth in culture filtrate supplemented media (CFSM) and media only, when using three sample types [bronchoalveolar lavage fluid (BALF), post-bronchoscopy (PB) sputum and induced sputum] in 22 EOT patients.

**PID**	**BALF**		**PB sputum**		**Induced sputum**		**ZN stain**	**BAP**	**Selecta**	**Recovered strain type**
	**CFSM**	**Media**	**CFSM**	**Media**	**CFSM**	**Media**				
250	-	-	3/3	1/3	2/3	1/3	NEG	POS	NEG	NA
253	1/3	-	2/3	3/3	3/3	1/3	POS	NEG	NEG	1 BEIJING
267	3/3	-	3/3	3/3	3/3	1/3	POS	NEG	POS	373 T1
273					1/3	1/3	NEG	NA	NA	NA
278	-	-	-	-	-	-	NA	POS	NEG	NA
291	^*^	^*^	1/3	1/3	3/3	1/3	NEG	POS	NEG	NA
300	1/3	-	2/3	1/3	3/3	1/3	NEG	POS	NEG	NA
306	-	-	1/3	-	3/3	2/3	NEG	POS	NEG	NA
314	1/3	-	-	1/3	-	-	NEG	POS	NEG	NA
315	1/3	1/3	1/3	1/3	3/3	3/3	NEG	POS	NEG	NA
318	1/3	1/3		1/3	2/3	2/3	NEG	POS	NEG	NA
334	-	-	-	-	3/3	2/3	NEG	POS	NEG	NA
332	-	-	-	-	-	-	NA	NA	NA	NA
335	1/3	1/3	1/3	1/3	1/3	1/3	NEG	POS	NEG	NA
338	3/3	1/3	2/3	3/3	1/3	2/3	POS	NEG	POS	1 BEIJING
351	-	-	-	-	1/3	2/3	NEG	POS	NEG	NA
357	-	-	-	-	-	-	NA	NA	NA	NA
359	-	-	1/3	-	1/3	-	NEG	POS	NEG	NA
365	1/3	-	-	1/3	1/3	1/3	NEG	POS	NEG	NA
375	-	-	-	-	1/3	2/3	NEG	POS	NEG	NA
382	-	1/3	2/3	1/3	1/3	1/3	NEG	POS	NEG	NA
392	-	-	1/3	-	1/3	-	NEG	POS	NEG	NA
% Positive	41	22.7	54.5	54.5	81.8	72.7				
% True positive	13.6	4.5	4.5	0	9	0				

*Recovered Mtb strain type was determined using spoligotyping. The true positives row indicates how many of the positive cultures were confirmed to be Mtb using confirmatory assays*.

*Growth is indicated as either positive in three of the replicate wells (3/3), two of the three replicates (2/3) or one of the three replicate wells (1/3) or no growth (-). Gray blocks indicate that a sample was not available*.

* *Indicates the wells became contaminated with fungal growth and confirmatory assays could not be conducted. The red blocks indicate the samples that were confirmed positive for Mtb*.

### An Altered Host Proteome Can Be Identified at the End of Treatment

Distinct FDG PET-CT responses at the end of treatment prompted us to investigate the host response at the site of disease using mass spectrometry-based proteomics. We analyzed BALF supernatant collected from a subset of the patients recruited in this study, namely five EOT patients, with a mixture of FDG PET-CT responses (Table 4) and five pretreatment active TB cases, using multiplexed proteomics with tandem mass tags (TMT 10 plex). This relative proteome comparison would enable us to gain fundamental insights into the host response at the site of disease during active TB and in clinically cured patients at the end of 6 month standard anti-TB therapy.

**Table 4:** End of treatment patients included for BALF shotgun proteomics analysis showing their FDG PET-CT score at month 6 [total glycolytic activity (TGAI), response pattern, intensity rank and cavity size], their differentially culturable tubercle bacilli (DCTB) status, and final outcome at EOT + 1 year.

**PID**	**PET score**					
	**TGAI**	**Response pattern**	**Intensity rank**	**Cavity**	**DCTB**	**Outcome**
250	620	Mixed	Very high	62.9	No	Cured
253	70.361	Resolved	Minimal	0	Yes	Cured
314	151.66	Improved	Mild	0	No	Cured
338	43,007	Mixed	Very high	39.9	Yes	Relapse
375	9562.6	Improved	Very high	1.8	No	Cured

We identified a total of 1,565 high confidence proteins in BALF from EOT and active TB cases, which contained more than two unique peptides and were identified at a 1% empirical protein false discovery rate (FDR). These proteins spanned six orders of magnitude in abundance (Figure S3). The most abundant protein was albumin, followed by several other proteins commonly identified in plasma, including serotransferrin, haptoglobin, alpha-1-acid glycoprotein, and alpha-2-macroglobulin. Importantly, we were able to probe the BALF proteome to a level where we could identify cytokines (interleukin-18, macrophage colony-stimulating factor 1 and macrophage inhibitory factor), chemokines (CXCL17), pulmonary surfactants (pulmonary surfactant-associated protein A, B, and D) and antimicrobial peptides (cathelicidin LL37 and neutrophil defensins) in these samples (Figure S3) identifying these low abundance proteins in clinical samples, especially biological fluids, is notoriously difficult. The proteins identified in BALF from EOT and active TB were mainly involved in processes associated with neutrophil activation, innate and adaptive immune responses, RNA metabolism and translation (Figure S4).

For quantitative analysis, the corrected reporter ion intensities for each sample was log 2 transformed, resulting in a normal distribution (Figures S5A–J). The data was further normalized by subtracting the median to center all distributions around zero and thereby minimize inter sample variation (Figure S5K). This data was used for further analysis. We were able to reproducibly quantify 1,521 proteins in all patient samples analyzed, after filtering out common contaminants such as albumin and keratin.

**Figure 3:**
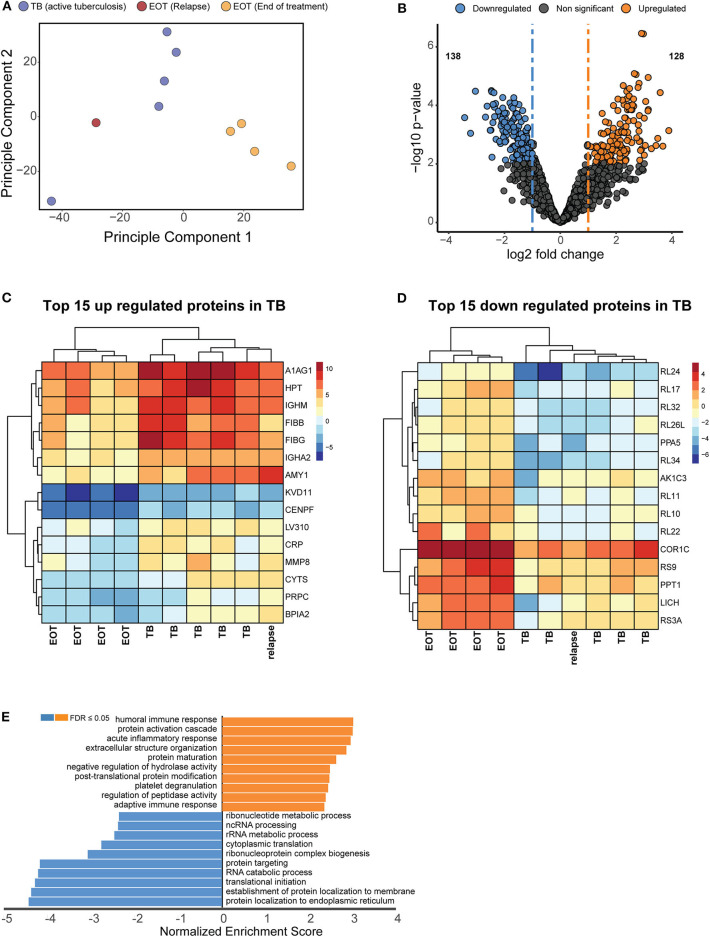
Unique proteome signatures exist in BALF from patients with active TB compared to patients maintaining clinical cure. **(A)** Principle component analysis of protein intensities obtained by mass spectrometry analysis of BALF from active TB patients at baseline (TB) and patients successfully completing 6 months anti-TB therapy (EOT). Active TB cases could be distinguished from the majority of EOT cases in principle component 1, with the exception of one EOT case. Follow up analysis of this patient revealed this was a relapse case with differentially culturable *M. tuberculosis*. **(B)** Volcano plot representing the differentially regulated proteins in active TB compared to EOT. The relapse sample was considered as a TB case for the statistical test. Heatmaps of the **(C)** top 15 upregulated and **(D)** downregulated proteins from the hypothesis testing. **(E)** Gene enrichment analysis (GSEA) of all significantly regulated proteins against gene ontology terms. GSEA was performed using the WebGestalt webserver (Liao et al., 2019).

Principle component analysis was used to identify the major sources of variation between the BALF proteomes of active TB and EOT cases, and to identify clusters in our data driven by protein expression profiles. As hypothesized, a clear separation was observed between the active TB and the EOT samples in the first component (Figure 3A). Interestingly, one EOT sample from this clinically cured set (PID 338) clustered with the active TB samples. During follow-up, this participant was subsequently diagnosed with recurrent TB disease (Figure 3A). Even though this participant completed 6 months of anti-TB treatment and was declared cured, a similar protein expression profile to that of an active TB case was observed 3 months before the participant returned to the clinic and was diagnosed as a relapse. To further confirm that the relapsed patient clusters with the TB cohort, we performed hypothesis testing by grouping the relapse as either active TB, EOT cure or removed from the sample set. We found that when relapse is added to the TB group there is an increase of 38 additional proteins (Figure S6) that distinguish active disease from cure. However, addition of the relapse case as an EOT cure resulted in little to no differential abundance of proteins due to the effects of the standard deviation which were introduced due to the active TB profile of the relapse distorting the EOT cured profiles. We therefore treated this sample as an active TB case for downstream analysis. We used hypothesis testing to determine the differentially regulated proteins between active TB and EOT cases. In total, 266 proteins were differentially regulated (two-fold change and adjusted *p*-value <0.05), where 138 proteins were significantly down regulated and 128 proteins were significantly up regulated during active TB disease (Figure 3B, Supplementary file 1). In the active TB cases, the top 15 regulated proteins by fold change included inflammatory and immune proteins such as CRP, BPIA2, and A1AG1 (ORM1) (Figure 3C). Furthermore, active TB was associated with increased levels of immunoglobulins, proteins associated with infiltration of neutrophils (MMP8, DEFA3, PRTN3), complement cascade members (C1QB, C1R, C1RL, C1S, C3, C4A, C5, C8A), and proteins playing a role in blood coagulation (F5, F13B, VWF, FGG, FGB, GP1BA). The top 15 down regulated proteins in the active TB cases were associated with translation as indicated by the presence of multiple ribosomal proteins (Figure 3D). To gain insights into the processes regulated between patients with active TB compared to those at the end of treatment (EOT), we performed gene set enrichment analysis on the differentially regulated proteins using non-redundant gene ontology terms. The greatest enrichment ratios were associated with proteins with increased abundance in the active TB cases, of which the humoral response and acute inflammatory response featured prominently (Figure 3E). On the other hand, proteins associated with RNA metabolic processes, protein synthesis and translation were more abundant in EOT cases.

## Discussion

Assessing true sterilization at the end of anti-TB therapy is paramount to reducing the global TB burden, especially since one of the highest priorities of the TB control programs is shortening treatment (World Health Organization, 2019). A key area of contention is the presence or absence of “persistent” Mtb cells in patients and whether this quiescent population is responsible for subsequent relapse after therapy has ceased (Zhang, 2014; Mandal et al., 2019). Here, we have shown that although patients at the end of successful anti-TB therapy are cured by clinical standards, we were able to identify a fraction of patients retaining DCTB at the end of treatment, two of which subsequently relapsed within the first year after stopping therapy. While the small sample size in this exploratory study does not have any statistical power, it is notable that Mtb growth was confirmed in CFSM from induced sputum of the two cases with the highest residual inflammatory burden on EOT FDG PET-CT, including the patient with subsequent relapse. This suggests that a correlation between the presence of DCTB and clinical outcome is plausible and that it should be further explored in prospective studies.

We decided to investigate both sputa and site of disease (BALF) for the presence of DCTB, as patients cannot always produce a sputum at the end of treatment. Using the DCTB assay at the end of treatment, most sputum sample types showed growth of commensal bacteria, even with routine decontamination procedures. Unsurprisingly, BALF was the optimal sample type for recovering differentially culturable bacteria, since BALF represents a direct representation of the site of disease, and with less exposure to commensal organisms. This has implications for the routine use of the DCTB assay for monitoring sterilizing cure from sputum in a clinical setting. While the lengthy and laborious nature of the DCTB assay would make large-scale implementation of the technique impractical, these results indicate that these DCTB cells are an important consideration when monitoring treatment response, specifically for treatment shortening studies. Viability assays measuring Mtb rRNA as a marker of treatment response currently show the most promise as alternatives to measure bacterial viability in patient samples (Honeyborne et al., 2011), and these should be tested in parallel to determine their ability to identify these differentially culturable bacilli.

The relapse rate observed in this study is consistent with rates seen in other studies, although we acknowledge the small number of cases (Sonnenberg et al., 2001; Luzze et al., 2013; Gillespie et al., 2014; Merle et al., 2014). Even though we cannot define the physiological state of the mycobacterial cells before recovery, this data shows that there remains a sub-population of cells that are undetectable by standard culture (MGIT), yet differentially culturable in the presence of CFSM. These results build on previous studies that have shown that DCTB can be detected in patient sputa from TB patients at baseline and early chemotherapy (Mukamolova et al., 2010; Chengalroyen et al., 2016; Dusthackeer et al., 2019) and that bacterial persistence is likely the cause of EOT inflammation (Malherbe et al., 2016). The fact that many more patients retain FDG avid lesions at EOT than have demonstrable DCTB may be due to a low number of viable bacteria or relative insensitivity of the DCTB assay. Further work is needed to examine the link between inflammation on PET-CT imaging and DCTB. Multiple studies have been necessary to examine this question especially when trying to correlate with relapse since only 4–10% of patients relapse. This study confirms and extends the previous studies by modifying the DCTB assay and by thoroughly examining the patients (PET-CT, BALF proteomics) specifically at the end of successful treatment. To our knowledge, this is the first study to investigate the presence of differentially culturable Mtb in sputum and BALF at the end of anti-TB therapy.

The patients that were identified as positive for DCTB yet have maintained cure indicate how the host immune response plays a significant role to curtail the remaining infection and direct treatment outcome. Indeed, although the factors responsible for relapse and reactivation have not yet been completely elucidated, immune suppression has a clear impact on this phenomenon (Connolly et al., 2007; Gideon and Flynn, 2011; Esmail et al., 2014; Manina et al., 2015). As we have shown previously, a large proportion of patients at the end of successful therapy show ongoing inflammation as detected by FDG PET-CT indicating patterns consistent with active disease (Malherbe et al., 2016), yet not all these patients will go on to relapse. In this study, we found that FDG PET-CT responses consistent with active TB only partially correlated with differentially culturable Mtb. One patient was positive for DCTB, yet FDG PET-CT indicated complete resolution of lesions. It must be noted that this patient sample was only positive for Mtb in the BALF sample in one of the triplicate wells, unlike the other patients, where Mtb was consistently detected in all samples analyzed. It would also be beneficial to assess how long these DCTB cells can be detected, as it is possible that although they are present at month 6, later time points would show that these have been cleared by the immune response. The heterogeneity in patient FDG PET-CT responses highlights how different lesions, even within the same host, can progress differently. It remains to be determined what factors determine whether a patient achieves true sterilization.

We therefore analyzed the global host proteome profiles of five end of treatment patients (consisting of one EOT patient that subsequently relapsed and four that had maintained cure with a mixture of FDG PET-CT responses) vs. active TB patients (before treatment) at the site of disease (BALF), to enhance our understanding of the lung microenvironment. Principal component analysis indicated distinct proteomic signatures stratifying active and clinically cured TB patients into distinct groups. Additionally, the relapsed participant, although standard sputum MGIT negative and clinically defined as cured at M6, produced a M6 BALF protein expression profile that clustered more closely with the active TB group than with the clinically cured group. Taken together, it is likely that patients who are prone to relapse can be detected within the BAL fluid, however further investigation is required with greater study cohorts.

Gene set enrichment analysis revealed that the major upregulated responses in active TB (pre-treatment samples) correspond to important immune responses notably, the acute inflammatory response and protein activation cascades. The acute phase response serves various important functions within the immune system, one of which is the activation of the complement cascade by positive acute-phase proteins (APPs) (Jain et al., 2011). Several APPs that are involved in the complement cascade were identified as upregulated in the BALF of active TB cases including CRP, C3, C5, and C1qrs pointing to classical and lytic activation pathways. The complement system is a key component of the innate immune response and is initiated rapidly upon infection to promote inflammation, recruit leukocytes, form a membrane attack complex and ultimately destroy invading pathogens (Ricklin et al., 2010). Although there are limited BALF proteomics studies in the context of TB, a previous report showed that BALF from healthy patients contains classical complement activity which leads to C3b binding of Mtb *in vitro* (Ferguson et al., 2004). Our findings are also consistent with proteomic investigations of blood-based signatures for TB progression, showing a similar elevation of members of the complement cascade, infiltration of neutrophils and blood coagulation during early disease (Scriba et al., 2017; Esmail et al., 2018; Penn-Nicholson et al., 2019), whilst providing additional information on altered pathways of treatment response. It stands to reason that following treatment and sterilizing cure, these pathways would return to lower levels. Unsurprisingly, we observed these processes in actively infected TB patients and their decrease in the M6 EOT clinically cured patients. Further characterization in a larger cohort of patients would be beneficial in assessing whether these proteomic signatures are consistent, as these results show promise in identifying distinguishing signatures of treatment response. If these observations can be extended, emphasis should be placed on replacing the invasive BALF procedure with a more easily obtained sample. Analysis of serum samples by multiplexed ion mobility spectrometry similarly shows a similar decrease of acute phase proteins in response to antibiotic treatment and culture status (Kedia et al., 2018). This supports the clinical translatability of identified BALF markers being applied for blood-based testing. To our knowledge, this is the first study to investigate host proteome changes at the site of disease during active TB and following anti-TB therapy. We were able to identify a large number of proteins spanning a broad dynamic range offering new avenues for targeted investigations.

## Conclusion

This study, although exploratory in nature, shows a comprehensive characterization of end of treatment patients from both a host and bacterial angle. We describe that DCTB can be detected at the end of successful anti-TB therapy and may represent a risk for relapse. Our methods were labor intensive and because of this they were limited in scope. Targeting dormant bacilli has been suggested as an important avenue to fight reactivation of TB and shorten treatment (Fattorini et al., 2013), and this study defines a pathway to further explore biomarkers of persistence in patients at the end of therapy.

## Data Availability Statement

The datasets presented in this study can be found in online repositories. The names of the repository/repositories and accession number(s) can be found in the article/Supplementary Material.

## Ethics Statement

The studies involving human participants were reviewed and approved by Medical Human Research Ethics Committee of Stellenbosch University (N10/01/013 and N16/05/070). The patients/participants provided their written informed consent to participate in this study.

## Author Contributions

CB, GW, SM, JW, and TH: study concept and design. SM: patient recruitment and visual scan analysis. TH, JG, and CB: acquisition, analysis, and interpretation of the proteomics data. CB and RV: acquisition of the microbiological data. CB: drafting of manuscript. CB, GW, TH, JG, SM, JW, RV, BK, NP, MT, and AL: manuscript revision and important intellectual content. All authors contributed to the article and approved the submitted version.

## Conflict of Interest

The authors declare that the research was conducted in the absence of any commercial or financial relationships that could be construed as a potential conflict of interest.
