# Supplementary material for: Investigating Non-sterilizing Cure in TB Patients at the End of Successful Anti-TB Therapy
Source: Front Cell Infect Microbiol. 2020 Aug 25;10:443. doi: 10.3389/fcimb.2020.00443 (PMC7477326; doi:10.3389/fcimb.2020.00443)
Supplement: Supplementary file 1 [file Data_Sheet_1.docx]

Supplementary Material

## Supplementary Figures


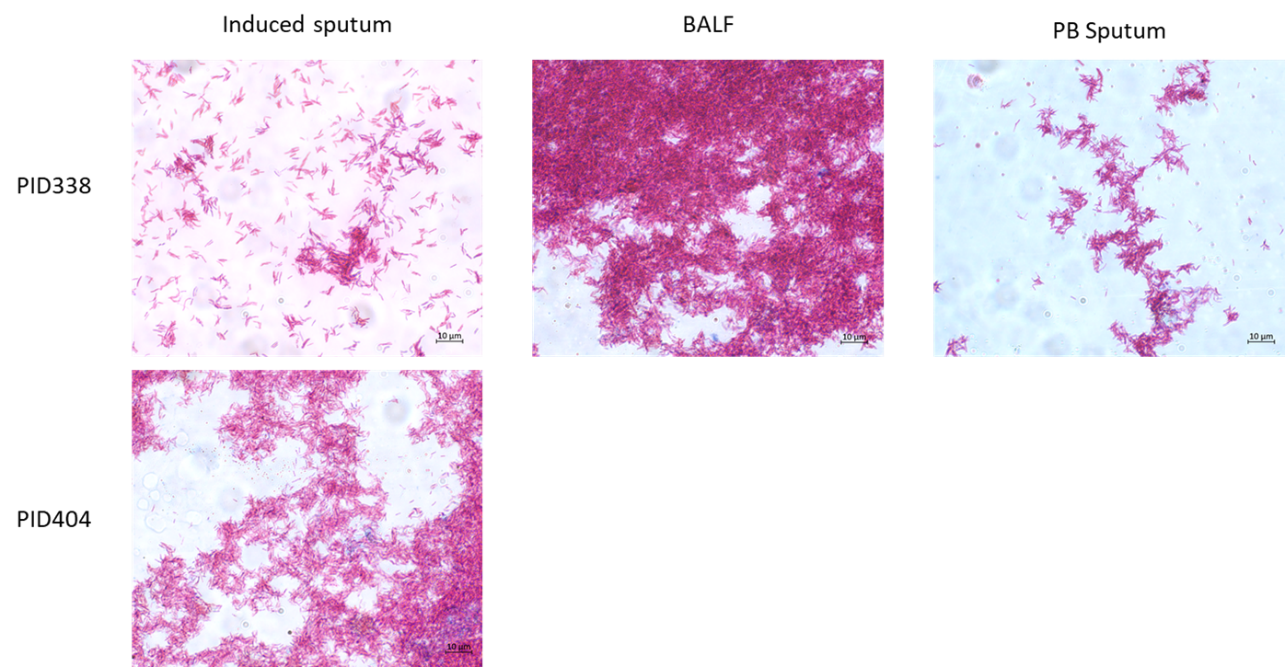


**Supplementary Figure S1.** Ziehl-Neelsen (ZN) stain of acid-fast bacilli showing purity of the recovered culture using the resuscitation assay in induced sputum (PID 338 and 404) and bronchoalveolar lavage fluid (BALF) and post-bronchoscopy sputum (PID338).

BALF

Induced sputum


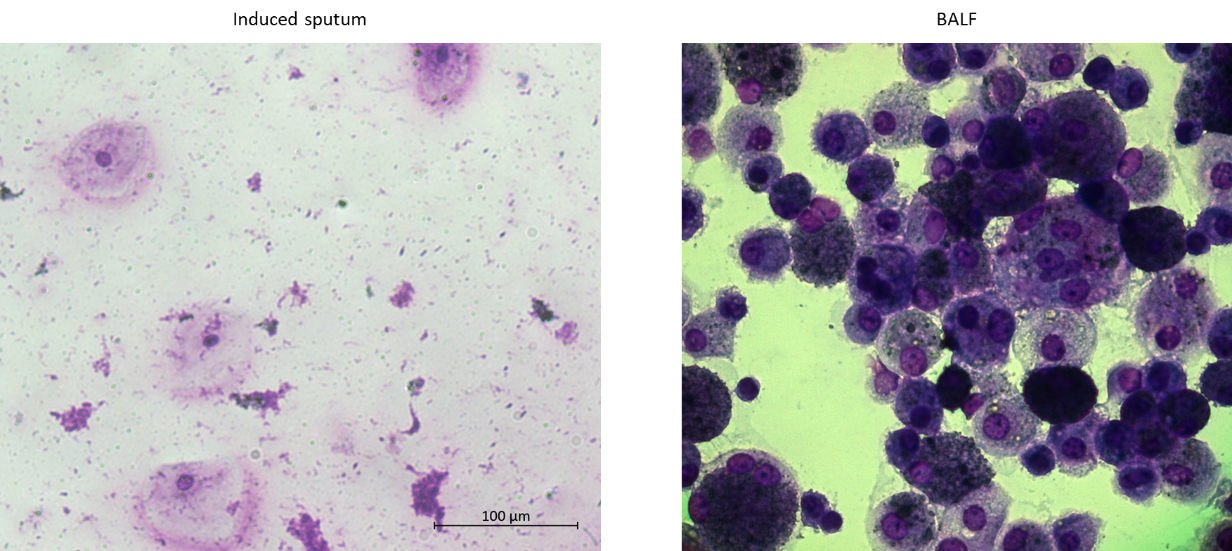


**Supplementary Figure S2.** Representative microscopy images of differential cell staining of an induced sputum (100X) and BALF sample (40X) from the same EOT patient. Samples were concentrated by cytocentrifugation at 95rpm for 7 minutes with slow acceleration and stained using Rapid-Diff stain.

.


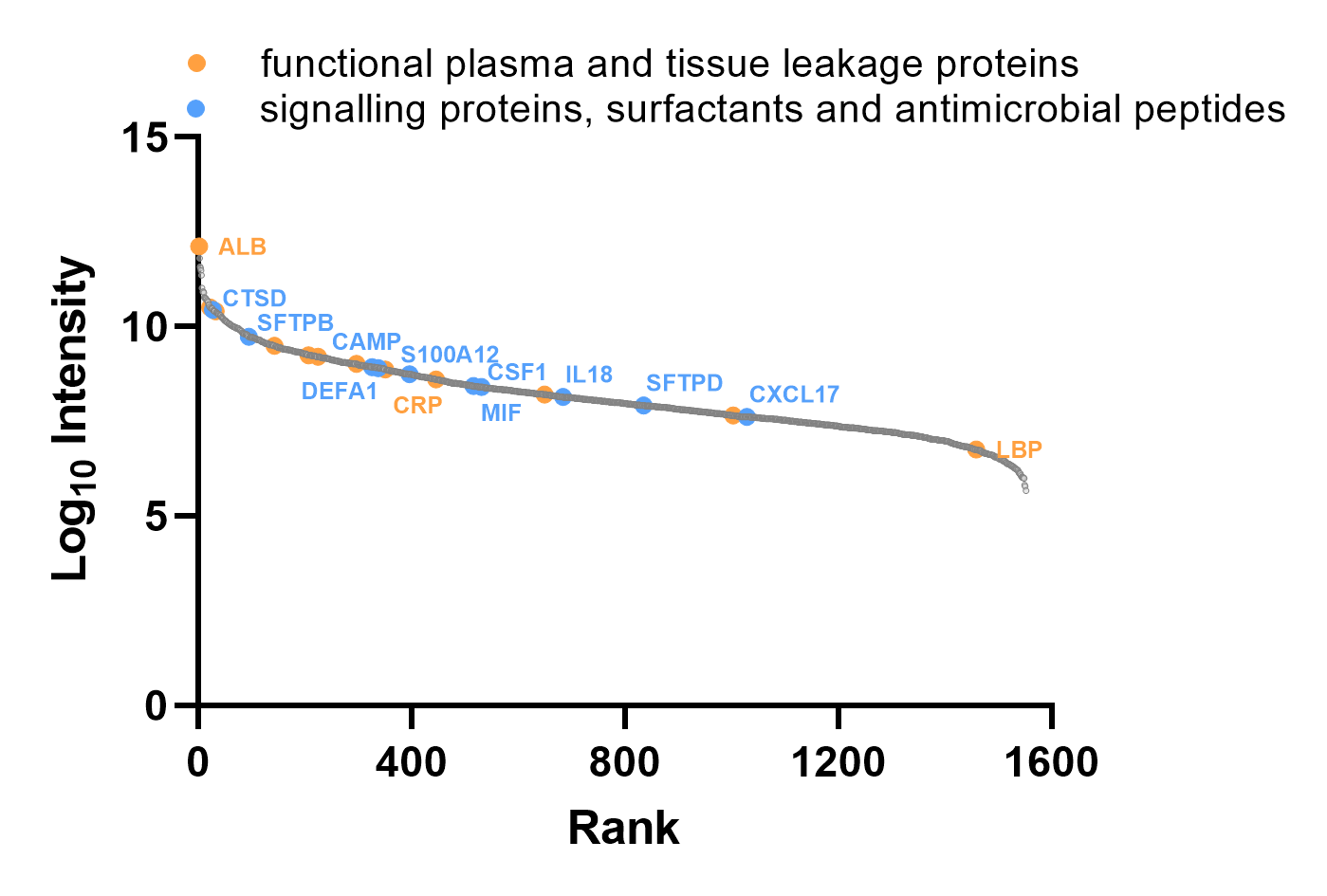


**Supplementary Figure S3.** Dynamic range estimation showing combined log_10_ intensity of the proteins identified in the study with functional plasma and tissue leakage proteins shown in orange and signaling proteins, surfactants and antimicrobial peptides shown in blue. The plot shows a dynamic range of 6 orders of magnitude.


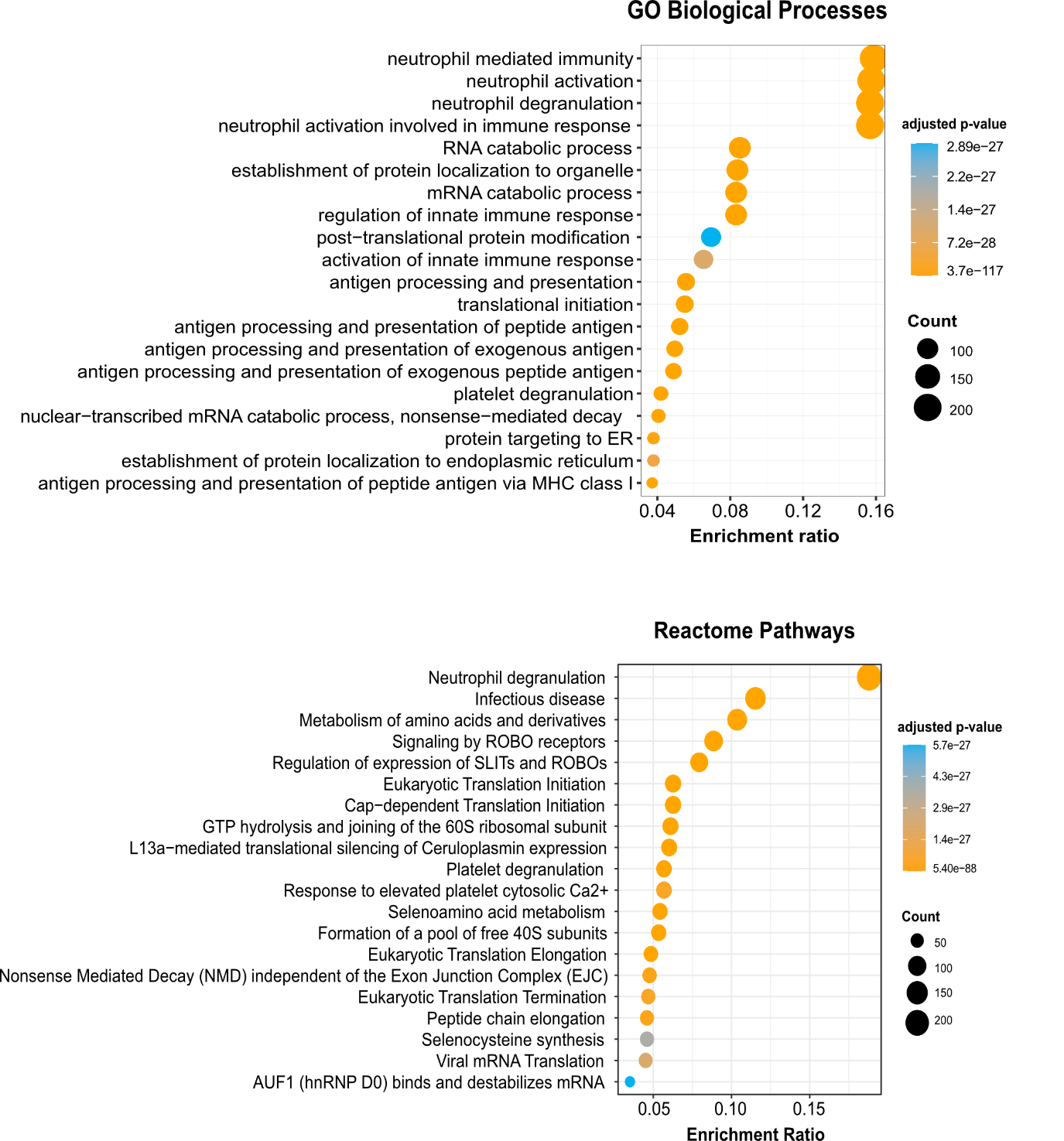


**Supplementary Figure S4.** Dotplot showing enrichment results of the GO biological processes and Reactome pathways. The size of the circle signifies the count of the biological process or reactome pathway. The colour denotes the adjusted p-value.


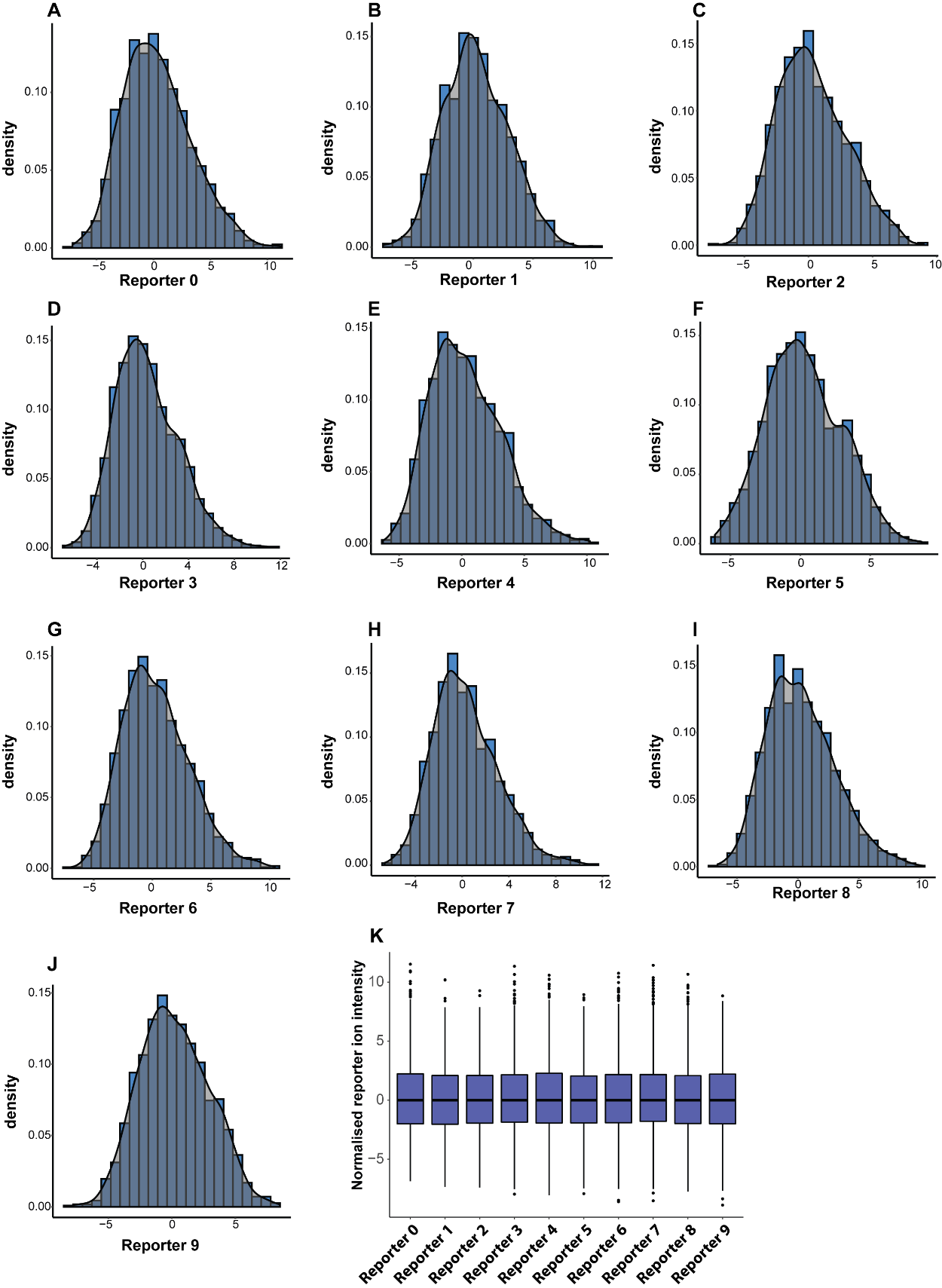


**Supplementary Figure S5.** **Quality control of reporter ion intensities after normalization.  A-J)** Histograms depicting the distribution of reporter ion intensities for each normalized reporter ion. **H)** Box and whiskers plot depicting the distribution of data for each reporter. Each reporter has been normalized to center the data around zero by subtracting the median. **K)** Boxplot representing reporter ion intensities across all samples after log transformation and normalization by subtracting the median. No major shifts in mean intensity occurs between reporters.


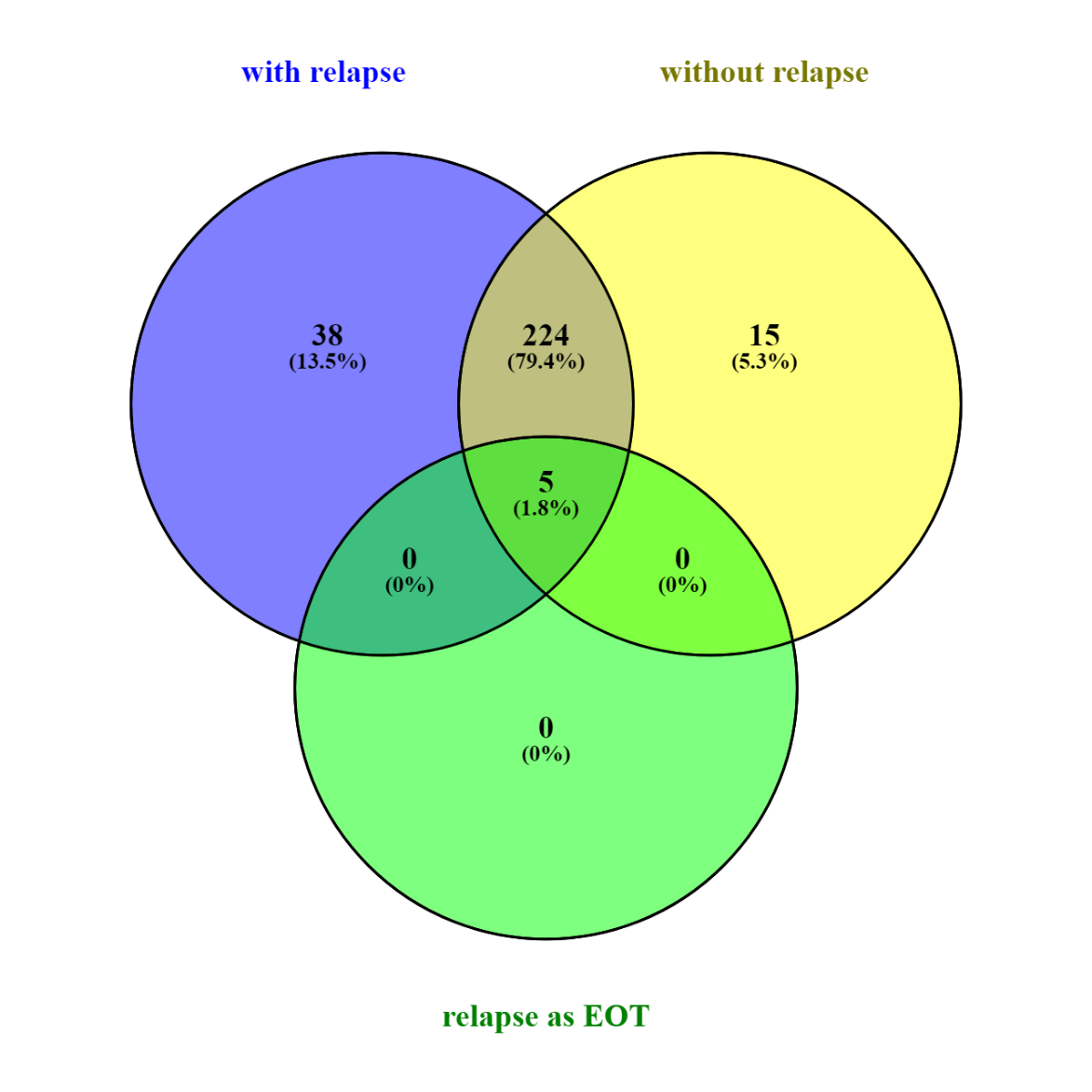


**Supplementary Figure S6.** Venn diagram showing the differential expression of proteins when analyzed with the EOT relapse (PID 338) case included as a TB case (with relapse), excluded from the analysis (without relapse) and included as an EOT case (relapse as EOT).

## Supplementary Tables

T**able S1:** Clinical and microbiological parameters of pulmonary TB patients (PID) assessed after successful anti-TB therapy (M6) using the Differentially culturable tubercle bacilli (DCTB) assay. Age in years, median (range), diagnosis (Dx), body mass index (BMI), chest x-ray (CXR), time to positivity (TTP), time to negativity (TTN), BALF (BLF), induced sputum (iS) post-bronchoscopy sputum (pbS).

| **PID** | **Age** | **Sex** | **HIV** | **Hba1c** | **Smoker** | **Previous TB** | **Treatment adherence (%)** | **BMI Dx** | **BMI M6** | **CXR M6** | **CXR M18** | **Culture Dx TTP (days)** | **Culture TTN (weeks)** | **DCTB** | **Source** | **Outcome** |
| --- | --- | --- | --- | --- | --- | --- | --- | --- | --- | --- | --- | --- | --- | --- | --- | --- |
| 250 | 43 | M | NEG | 6,1 | Daily | NO | 100 | 16,8 | 17,8 | Improved | Improved | 8 | 8 |  |  | Cured |
| 253 | 29 | M | NEG | 5,9 | Daily | NO | 100 | 19,2 | 21,3 | Worse | Improved | 11 | 16 | POS | BLF | Cured |
| 267 | 57 | M | NEG | 6 | EX | YES | 100 | 18,2 | 21 | No change | No change | 6 | 16 | POS | BLF/iS | Cured |
| 273 | 32 | M | POS | 5,7 | Daily | NO | 100 | 18,1 | NA | Improved | Improved | 7 | 8 |  |  | Cured |
| 278 | 36 | M | NEG | 5,4 | NO | YES | 99 | 20,8 | 21,6 | Improved | No change | 7 | 8 |  |  | Cured |
| 291 | 33 | M | NEG | 5 | Daily | NO | 99 | 16,6 | 19,4 | No change | Improved | 5 | 24 |  |  | Cured |
| 300 | 40 | M | NEG | 5,3 | Daily | NO | 100 | 20 | 20,2 | Improved | No change | 4 | 2 |  |  | Cured |
| 306 | 55 | M | NEG | 5 | Daily | NO | 100 | 20,9 | 21,2 | No change | Improved | 5 | 8 |  |  | Cured |
| 314 | 42 | F | NEG | 5 | Daily | NO | 100 | 22,6 | 24,2 | Improved | No change | 16 | 8 |  |  | Cured |
| 315 | 26 | F | NEG | 5,6 | Daily | NO | 100 | 17 | 17,4 | Improved | No change | 4 | 24 |  |  | Cured |
| 318 | 31 | F | NEG | 5,9 | NO | NO | 100 | 21,5 | NA | Improved | Improved | 16 | 16 |  |  | Cured |
| 332 | 58 | M | NEG | 6,1 | Daily | NO | 100 | 21 | 23,9 | Improved | NA | 5 | 8 |  |  | Cured |
| 334 | 39 | M | NEG | 5,9 | EX | YES | 100 | 16,1 | 16,3 | Improved | Worse | 9 | 4 |  |  | Cured |
| 335 | 49 | M | NEG | 5,9 | Daily | YES | 100 | 15,4 | 17,2 | Improved | No change | 5 | 8 |  |  | Cured |
| 338 | 48 | M | NEG | 5,3 | Daily | YES | 99 | 16 | 16,5 | No change | No change | NA | 8 | POS | BLF/iS/pbS | Relapse |
| 351 | 22 | F | NEG | 5,5 | Daily | NO | 100 | 17,9 | 20,9 | Improved | Improved | 5 | 8 |  |  | Cured |
| 354 | 62 | F | NEG | 6 | Daily | YES | 100 | 27,4 | 29,8 | Improved | Improved | 6 | NA |  |  | Cured |
| 357 | 51 | M | NEG | 5,2 | Daily | YES | 100 | 18,7 | 18,4 | Improved | No change | NA | NA |  |  | Cured |
| 359 | 48 | F | NEG | 5,5 | Daily | YES | 100 | 22,1 | 23 | Improved | No change | 16 | 2 |  |  | Cured |
| 365 | 65 | M | NEG | 5,2 | < daily | YES | 100 | 20,6 | 20,5 | Improved | No change | NA | NA |  |  | Cured |
| 370 | 26 | F | NEG | 5,2 | < daily | NO | 100 | 16,5 | 18,6 | Improved | No change | 4 | 8 | POS | iS | Cured |
| 375 | 54 | M | NEG | 6,3 | Daily | NO | 100 | 15,2 | 17,8 | Improved | Improved | 10 | 8 |  |  | Cured |
| 382 | 43 | F | NEG | NA | Daily | NO | 99 | 17,2 | 18,7 | Improved | Improved | 4 | 16 |  |  | Cured |
| 392 | 21 | M | NEG | NA | Daily | NO | 100 | 17,5 | 17,9 | Improved | No change | 11 | 8 |  |  | Cured |
| 393 | 39 | M | NEG | NA | Daily | NO | 96 | 16,3 | 17,9 | Improved | Improved | NA | 16 |  |  | Cured |
| 394 | 44 | F | NEG | NA | Daily | YES | 100 | 25,6 | 26,8 | Improved | Improved | NA | NA |  |  | Cured |
| 396 | 52 | M | NEG | NA | Daily | NO | 100 | 20,1 | 22,5 | No change | Improved | 5 | 8 |  |  | Cured |
| 397 | 47 | M | NEG | NA | Daily | YES | 96 | 20,3 | 19,6 | No change | Improved | NA | NA |  |  | Cured |
| 404 | 49 | F | NEG | NA | NO | YES | 99 | 16,6 | 15,6 | No change | No change | 8 | 24 | POS | iS | Relapse |
| 413 | 29 | M | NEG | NA | Daily | NO | 100 | 18,5 | NA | Improved | Improved | 8 | 4 |  |  | Cured |
| 426 | 33 | F | NEG | NA | NO | NO | 100 | 22 | NA | Improved | Improved | 9 | 8 |  |  | Cured |
| 425 | 30 | M | NEG | NA | Daily | NO | 100 | 19,2 | NA | Improved | Improved | 13 | 4 |  |  | Cured |
| 422 | 57 | M | NEG | NA | Daily | NO | 100 | 23,3 | NA | Improved | Improved | 16 | 2 |  |  | Cured |
| 427 | 51 | M | NEG | NA | Daily | NO | 99 | 17,2 | NA | Improved | Improved | 9 | 4 |  |  | Cured |
| 428 | 32 | M | NEG | NA | Daily | NO | 100 | 15 | NA | Improved | Improved | 7 | 8 |  |  | Cured |
| 429 | 52 | M | NEG | NA | Daily | YES | 100 | 15,9 | NA | Improved | Improved | 6 | 8 |  |  | Cured |
| 430 | 45 | M | NEG | NA | Daily | NO | 100 | 18,6 | NA | Improved | Improved | 13 | 8 |  |  | Cured |
| 431 | 23 | F | NEG | NA | < daily | NO | 100 | 20,4 | NA | Improved | Improved | 5 | 16 |  |  | Cured |
| 390 | 54 | F | NEG | NA | NO | NO | 99 | 21,4 | 19,3 | Improved | Improved | 11 | 8 |  |  | Cured |
| 437 | 23 | F | NEG | NA | Daily | NO | 100 | 20,8 | NA | Improved | Improved | 5 | 4 |  |  | Cured |
| 433 | 31 | M | NEG | NA | Daily | NO | 100 | 26,1 | NA | Improved | Improved | 8 | 2 |  |  | Cured |
| **Total 41** | **43 (21-65)** | **27 Male 66%** | **40 HIV Negative 97.5%** |  | **34 Smoker 82.93%** | **13 Previous TB 31.7%** | **>99%** | **19 (15-27.4)** |  | **33 Improved 80%** | **26 Improved 63%** | **7 (4-16)** | **8 (2-24)** | **5 DCTB Positive 12.2%** |  | **2 relapse 4.88%** |

**Table S3:** Proteomics analysis data uploaded as excel sheet (supplementary table proteomics data) showing identified proteins with Uniprot ID and corresponding unique peptide and reporter ion information and analysis of differential regulation (significance *p* < 0.05).
